# A simple method to estimate the in-house limit of detection for genetic mutations with low allele frequencies in whole-exome sequencing analysis by next-generation sequencing

**DOI:** 10.1186/s12863-020-00956-x

**Published:** 2021-02-18

**Authors:** Takumi Miura, Satoshi Yasuda, Yoji Sato

**Affiliations:** 1 Division of Cell-Based Therapeutic Products, National Institute of Health Sciences, 3-25-26 Tonomachi, Kawasaki-ku, Kawasaki, 210-9501 Japan; 2 Department of Quality Assurance Science for Pharmaceuticals, Graduate School of Pharmaceutical Sciences, Nagoya City University, 3-1 Tanabe-dori, Mizuho-ku, Nagoya, 467-8603 Japan; 3 Department of Cellular & Gene Therapy Products, Graduate School of Pharmaceutical Sciences, Osaka University, 1-6 Yamadaoka, Suita, Osaka, 565-0871 Japan; 4 Department of Translational Pharmaceutical Sciences, Graduate School of Pharmaceutical Sciences, Kyushu University, 3-1-1 Maidashi, Higashi-ku, Fukuoka, 812-8582 Japan

**Keywords:** Limit of detection, Next-generation sequencing, Allele frequency, Omics research

## Abstract

**Background:**

Next-generation sequencing (NGS) has profoundly changed the approach to genetic/genomic research. Particularly, the clinical utility of NGS in detecting mutations associated with disease risk has contributed to the development of effective therapeutic strategies. Recently, comprehensive analysis of somatic genetic mutations by NGS has also been used as a new approach for controlling the quality of cell substrates for manufacturing biopharmaceuticals. However, the quality evaluation of cell substrates by NGS largely depends on the limit of detection (LOD) for rare somatic mutations. The purpose of this study was to develop a simple method for evaluating the ability of whole-exome sequencing (WES) by NGS to detect mutations with low allele frequency. To estimate the LOD of WES for low-frequency somatic mutations, we repeatedly and independently performed WES of a reference genomic DNA using the same NGS platform and assay design. LOD was defined as the allele frequency with a relative standard deviation (RSD) value of 30% and was estimated by a moving average curve of the relation between RSD and allele frequency.

**Results:**

Allele frequencies of 20 mutations in the reference material that had been pre-validated by droplet digital PCR (ddPCR) were obtained from 5, 15, 30, or 40 G base pair (Gbp) sequencing data per run. There was a significant association between the allele frequencies measured by WES and those pre-validated by ddPCR, whose *p-*value decreased as the sequencing data size increased. By this method, the LOD of allele frequency in WES with the sequencing data of 15 Gbp or more was estimated to be between 5 and 10%.

**Conclusions:**

For properly interpreting the WES data of somatic genetic mutations, it is necessary to have a cutoff threshold of low allele frequencies. The in-house LOD estimated by the simple method shown in this study provides a rationale for setting the cutoff.

## Background

Compared with conventional Sanger sequencing, next-generation sequencing (NGS) technologies have enabled a more rapid and easier assessment of variants/mutations across the entire genome [1–3]. Therefore, NGS technologies have largely replaced microarray-based methods in many omic studies. Additionally, this extremely powerful technology for detecting genetic variations has caused a paradigm shift in medicine and has the potential for use in precision medicine (also known as “personalized medicine” or “genomic medicine”) based on individual genetic risk to disease [4, 5]. In the past few years, NGS techniques have been used for non-invasive prenatal testing to analyze fetal aneuploidy [6]. They are also utilized for various other applications, including the detection of mutations in tumor-related genes for quality control of cell therapy products or genetically modified cell products [7, 8]. However, the limit of detection (LOD) of NGS-based techniques has not been well defined. Despite the increasing use of NGS for detecting low-level somatic mutations in clinical tissue samples and cell substrates for biopharmaceuticals, including cell therapy products and genetically modified cell products, the LOD of NGS for allele frequencies (AFs) is not verified often in each laboratory. Moreover, prior to detecting mutations with low AFs, researchers should have a cutoff threshold to eliminate false-positive results, using the determined or estimated LOD based on a clear understanding of the performance and limitations of their NGS-based methodology in their laboratories.

Currently, three types of NGS-based analytical methods are mainly used to identify genomic mutations: (i) whole-exome sequencing (WES), (ii) whole-genome sequencing (WGS), and (iii) targeted sequencing (TS) [9, 10]. For example, to detect germline mutations, the commonly required depths of sequencing are usually 75–100× and 30–50× for WES and WGS, respectively [11], where × indicates the fold number of unique reads that include a given nucleotide in the reconstructed sequence. In contrast, to analyze somatic mutations, deeper sequencing must be performed because unlike germline mutations, somatic mutations are mosaic [12], and therefore genetically abnormal cells with somatic mutations can be present in very low numbers relative to normal cells [13]. Most TS methods have an average depth of coverage of 500× or more [14]. Therefore, in contrast to WES and WGS, TS can guarantee high analytical sensitivity and specificity for detecting single-nucleotide variants (SNVs), indels, and selected translocations [15, 16]. In addition, TS is more rapid and cost-effective than are WES and WGS. However, its limitation is that only mutations currently known to be important can be detected. WES can be useful as a comprehensive approach to identify pathogenic variants in protein-coding regions of the genome. Moreover, with decreasing sequence costs (because of sequencing platforms), analysis using WES data is an effective approach for identifying more causal mutations than detecting mutations by TS.

Currently, several studies have reported the application of WES focusing on the exome using NGS techniques to diagnose some undiagnosed diseases [17] or to control the quality of cell substrates for biopharmaceuticals [18, 19]. Therefore, to accurately evaluate the relationship between genetic mutations and diseases or the quality of cell substrates, the in-house performance of WES should be carefully evaluated in advance. Otherwise, the results obtained by WES would greatly affect decision-making in clinical or research settings. Thus, we aimed to develop a simple method to estimate the LOD for genomic variants with low AFs, which is required in setting a cutoff threshold in WES.

## Results

### Sequencing coverage of on-target regions

**Fig. 1:**
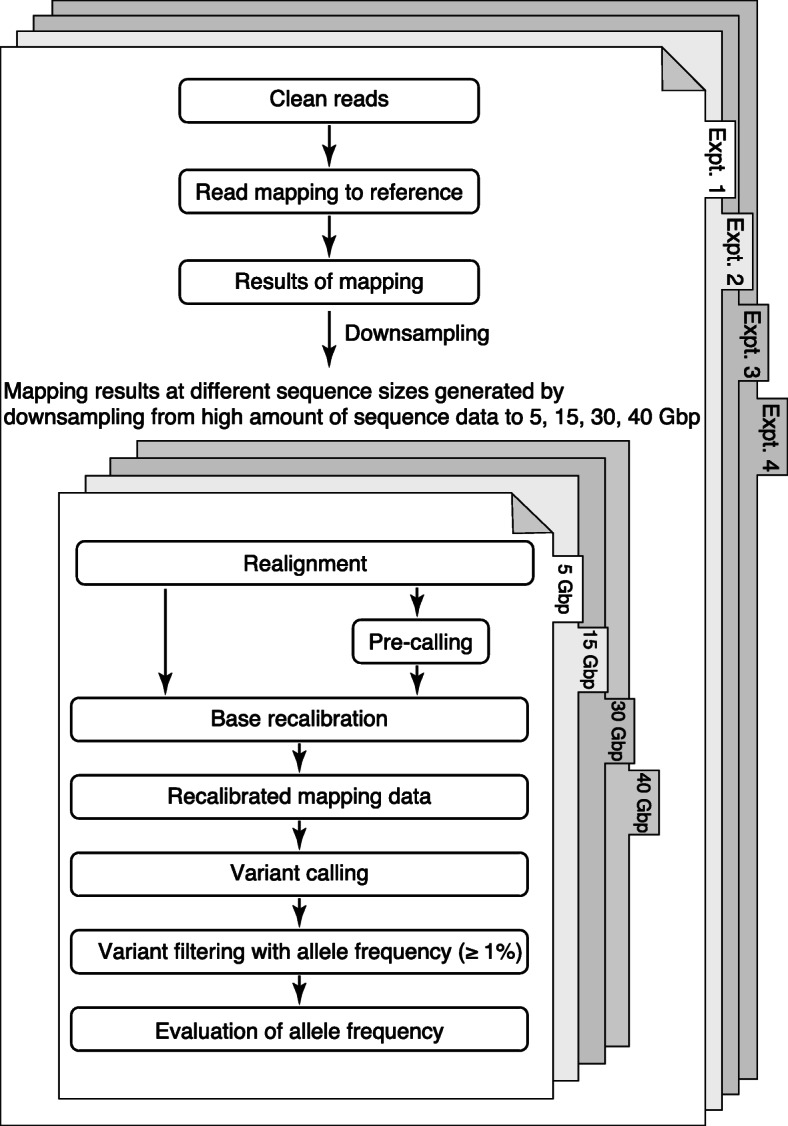
Experimental overview of analysis strategies used in this study. A reference material was sequenced using the same whole-exome sequencing (WES) process in quadruplicate technical replicate experiments. Expt., experiment; Gbp, giga base pairs

To assess the performance of a measurement system for AFs of genomic variants by NGS, we carried out a performance test for NGS using genomic DNA with 20 mutations, whose AFs had been determined by digital droplet PCR (ddPCR) in advance, as a reference material for NGS. After performing WES and mapping to the reference sequence (hg19), we investigated the relationship between the precision of the measured AFs and the amount of sequencing data. Four sequencing datasets with different sizes [5, 15, 30, and 40 G base pairs (Gbp)] were obtained by randomly downsampling all sequencing data obtained by mapping. Next, mutations such as SNVs in each sequencing data size were detected by genome-wide analysis. In our workflow strategy, we performed independent quadruplicate technical replicate experiments, which included the whole procedure starting from library preparation (Fig. 1), using the pre-validated genomic DNA sample to characterize the detection of AFs by WES. The results showed that the average numbers of mapped reads per experiment were 50 million at a data size of 5 Gbp, 150 million at 15 Gbp, 299 million at 30 Gbp, and 399 million at 40 Gbp (Table S1). Of the reads mapped to the genome, approximately 76% was mapped to the on-target region in all analyses, indicating that the sequencing data were acquired uniformly. The average value of the mean depth per experiment was 63× at 5 Gbp, 189× at 15 Gbp, 377× at 30 Gbp, and 503× at 40 Gbp. In addition, the cover rate per experiment, which was achieved from whole exome capture and sequencing, was more than 91% with ≥20× of the depth at 5 Gbp, and more than 96% with ≥40× of the depth at 15, 30 and 40 Gbp. The complete coverage information regarding the differences in all other sequence experiments is presented in Table S1.

### Measurement of reproducibility of AFs

**Fig. 2:**
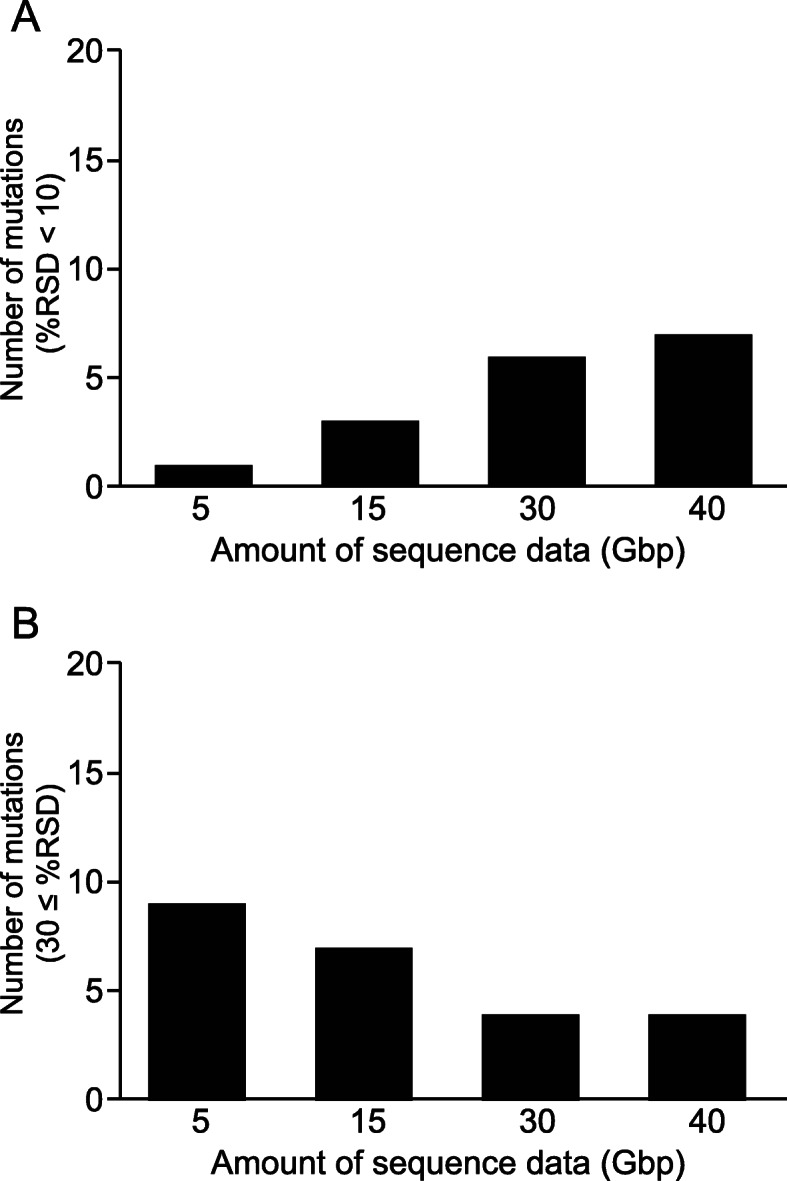
Relationship between the relative standard deviation (RSD) values for allele frequencies (AFs) obtained from WES analysis and the sequencing data size. The RSD values (%) for AFs of the 20 genetic mutations were calculated based on the data obtained from quadruplicate technical replicates. As the size of sequencing data increased, the number of mutations with RSD values < 10% increased (**a**) while those ≥30% decreased (**b**)

To assess the reproducibility of the WES-based AF measurement of genetic mutations, we next investigated whether point mutations in different genomic regions can be detected with high precision in quadruplicate technical replicate experiments at each sequencing data size (5, 15, 30, and 40 Gbp). The pre-validated genomic DNA employed as reference material contained 20 mutations with AFs of 1.0–33.5%, which is particularly suitable for the evaluation of a WES system to measure mutations with low AFs. By using these 20 mutations, we examined the performance of WES for mutation analysis, based on the % relative standard deviation (%RSD) value as the degree of variability of AFs. %RSD values for AFs of the 20 genetic mutations were calculated using the data from the quadruplicate technical replicates of the reference genomic DNA. As shown in Fig. 2, with an increasing amount of sequencing data, the number of mutations with an RSD < 10% and ≥ 30% increased and decreased, respectively, indicating that the larger the sequencing data size, the better the repeatability/precision of the WES system.

### Measurement accuracy of AFs

**Fig. 3:**
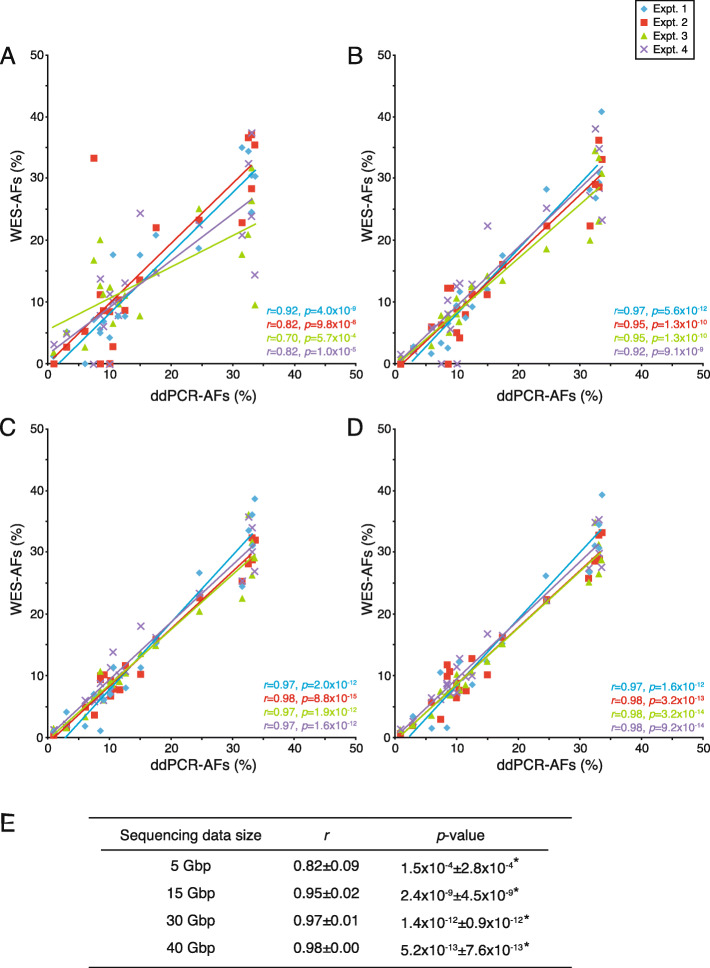
Correlation of allele frequencies validated by WES and droplet digital PCR (ddPCR) analysis. The degree of correlation of the 20 genetic mutations detected in the reference material across quadruplicate technical replicate experiments (Expt. 1–4) was assessed by linear regression analysis. The combination (ddPCR-AFs vs. WES-AFs) for regression analysis is displayed in each sequencing data size of 5 (**a**), 15 (**b**), 30 (**c**), and 40 (**d**) Gbp. In the graphs, each dot color represents one of the four technical replicates. **e** The relationships between experimentally detected WES-AFs and pre-validated ddPCR-AFs were verified by Pearson’s correlation coefficient (*r*). The *p*-value of the correlation was determined using the *t*-distribution. Mean values [± standard deviation (SD)] of the *r* and *p*-values obtained from four experiments were calculated for each sequencing data size (*P < 0.05)

The LOD of WGS/WES-based analysis for somatic genomic variants with low AFs must be as low as possible, preferably between 1 and 10% or less. However, the LOD for such genetic mutations by NGS can vary widely depending on the sequencing coverage. Therefore, we examined the relationship between the LOD and sequencing coverage in our NGS-based analysis system. Based on each sequencing data size, which was derived from the quadruplicate technical replicate experiments, the WES-based AFs (WES-AFs) of each of the 20 mutations were obtained. Overall, these WES-AFs were significantly associated with the AFs pre-validated by ddPCR (ddPCR-AFs; Fig. 3a–e), which demonstrates the accuracy of the WES-AFs. Therefore, the arithmetic mean values of WES-AFs obtained from the multiple technical replicates were used as the representative values for further analysis.

### LOD of AFs

**Fig. 4:**
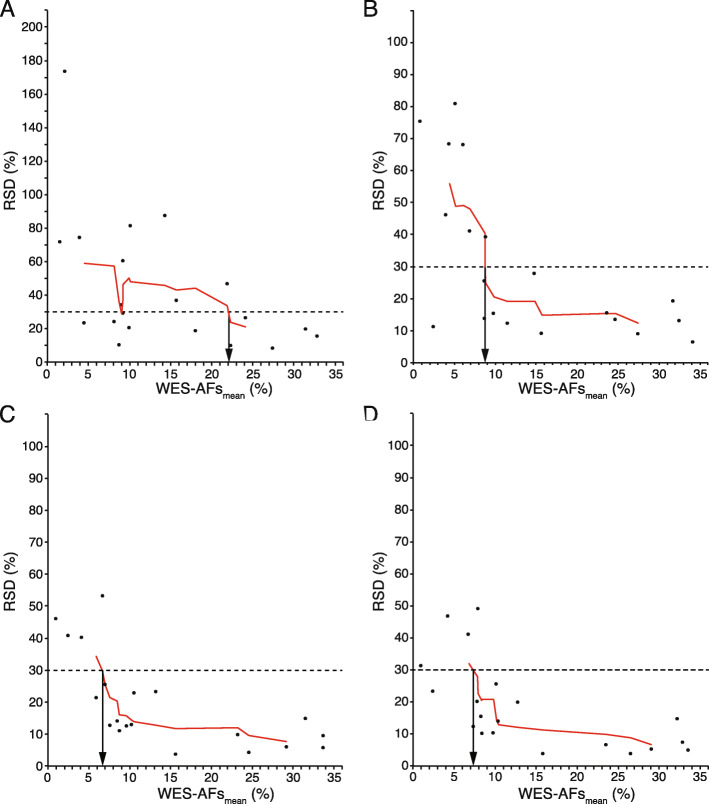
Limit of detection (LOD) of allele frequency in WES analysis. The %RSD values of AFs calculated from independent quadruplicate experiments were plotted against the mean values of AFs (mean of WES-AFs) obtained from their quadruplicate replicates. The trend in the consistency for %RSD vs. mean values of WES-AFs is represented by the 7 point-moving average curve on the graph. The derivation of LOD_30%RSD_ is illustrated by dotted lines (see arrow). All analyses were performed using the following sequencing data sizes: 5 (**a**), 15 (**b**), 30 (**c**), and 40 (**d**) Gbp

When the %RSD values of the 20 mutations derived from the quadruplicate technical replicates at different sequencing data sizes were plotted against their mean WES-AFs, the plots showed large fluctuations (Fig. 4a–d). Therefore, to obtain a clearer understanding of the relationship between the mean WES-AFs and %RSD values, we visualized and interpreted the data using a moving average of %RSD values. The moving average was calculated as a mean of 3, 5, or 7 adjacent data points of %RSD values. As shown in Fig. 4a–d and Fig. S1A–S1D, all moving average curves at different sequencing data sizes showed a trend where %RSD decreased as the mean WES-AF increased, allowing objective evaluation of measurement error variations of the determined AFs.

**Fig. 5:**
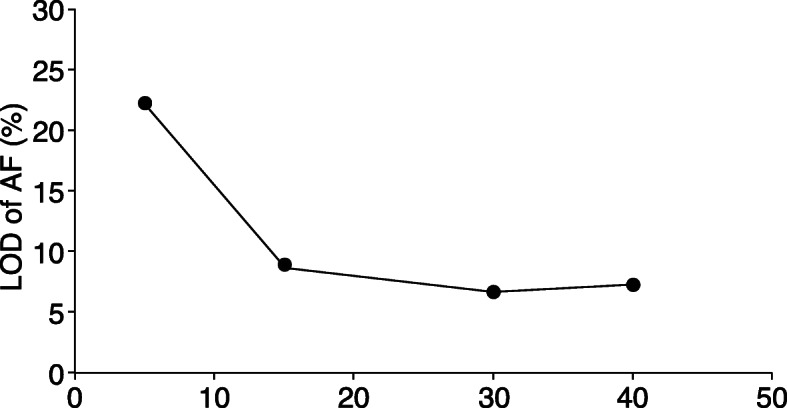
Assessment of AF LOD in WES analysis. Line graph showing the trend in correlation between LOD and sequencing data size (from Fig. 4). Even when WES was performed using a sequencing data size > 15 Gbp, the LOD remained relatively constant

In the present study, the LOD was defined as a WES-AF where its mean value was 3.3 times higher than its own standard deviation [20, 21], resulting in an RSD of 30% [22, 23]. Therefore, when the mean values of WES-AF with an RSD of 30% were read from the moving average curve graphs in Fig. 4a–d, the AFs in the sequencing data sizes of 5, 15, 30, and 40 Gbp were 22.1, 8.7, 6.6, and 7.2%, respectively (Fig. 5).

Separately, to examine the influence of the method used for DNA library preparation in our study, we also performed WES analysis using the Illumina TruSeq Exome Enrichment Kit for library preparation. As shown in Fig. S2A–S2D, AFs in the sequencing data sizes of 15, 30, 40 Gbp were 7.3, 6.3 and 6.2% at an RSD value of 30%, respectively, indicating comparable LOD values to those in Fig. 5. Therefore, even if another library preparation method is employed, there is not much difference in estimated LOD when the sequencing data size is ≥15 Gbp. These results support the reproducibility of our method for estimating LOD—regardless of library preparation method—as well as the reproducibility of the RSD value trend derived from the technical replicate experiments.

## Discussion

In the present study, we employed a simple method for obtaining an appropriate in-house cutoff to evaluate genetic mutations with low AFs in WES by estimating the LOD based on WES-AF %RSDs, which were derived from multiple technical replicates of a reference genomic DNA sample. The AFs of the 20 mutations in the reference sample were pre-validated using ddPCR by the manufacturer, which was also useful for evaluating the accuracy of WES-based analysis. To detect mutations with low AFs, the WES-based analysis system must exhibit high measurement reproducibility/precision. Therefore, we examined the depth (sequencing data size) necessary for the detection of such mutations. We repeatedly analyzed the reference genomic DNA sample under the same conditions to compare the AFs of the mutations detected in each WES and evaluated the degree of variation (Fig. 1). As shown in Fig. 2, the %RSDs of the WES-AFs decreased when the sequencing data size increased. Therefore, the lower the LOD of WES-AFs, the larger the sequencing data size that is required for AF measurement; this is because the LOD can be defined as a WES-AF whose mean value is 3.3 times higher than its own standard deviation, resulting in an RSD of 30% [22, 23]. The relationship between the average WES-AF and its %RSD value of quadruplicate technical replicates was evaluated by creating scatter plots of the %RSD values against the average values of each WES-AF and by drawing a moving average curve based on these scatter plots; accordingly, a decreasing trend of %RSD value was identified as the average WES-AF value increased (Figs. 4 and S1). Therefore, based on the definition described above and the smoothed moving average curve of the %RSD value, the LOD values of WES-AF can be easily estimated. As shown in Figs. 5 and S2D, the LOD value did not decrease linearly with an increase in sequencing data size; however, it almost reached its lowest value at 15 Gbp or more, indicating that the WES designed in this study requires a sequencing data size of ≥15 Gbp to detect mutations with a low AF of 5–10%, regardless of library preparation methods, and that increasing the sequencing data size (> 15 Gbp) does not markedly contribute to further decreasing the LOD. Another advantage of our approach is that the LOD can be estimated even when a pre-validated reference material is not available. In such cases, the mean AFs of specific mutations and their RSD values, obtained from multiple technical replicates of NGS analysis for any genomic DNA sample, can be used as a substitute for reference genomic DNA.

In NGS-based genetic mutation analysis, the number of cells (genomic copy number) targeted for analysis is another factor to be considered. One sequenced read can be considered to correspond to one genome molecule (one copy). In addition, as genomic DNA on autosomes is typically present as two copies per somatic cell, it can be assumed that two reads are derived from one cell. Since the reads to be mapped are often prepared from genomic DNA randomly selected from a large number of cells, two reads are not necessarily derived from one cell. However, because of the limited number of reads mapped, the genomes of all the cells used in library preparation are not covered. For example, in the target sequence, the number of reads (depth) is 1000–10,000× at most [11]. Approximately 6.6 pg of genomic DNA can be extracted from a diploid primary cell (3000 ng of genomic DNA was used to create a library for exome sequencing in this study), which corresponds to 4.5 × 10^5^ cells. Considering that there is an upper limit on the number of reads to be mapped, the applications at clinical or research settings require a depth of > 1000–10,000× coverage even for the target sequence. Thus, regardless of the read depth in exome analysis, it is not possible to analyze all 4.5 × 10^5^ cells. Therefore, to identify false-positive mutations, it is extremely important to evaluate the LOD of individual analysis systems and assign a cutoff threshold for NGS devices, which can be determined using a reference material containing accurately validated genetic mutations.

In summary, using our method, the LOD for AFs in exome sequencing was between 5 and 10% when the sequencing data size was 15 Gbp or more. The LOD may depend on various conditions, including the method of sample preparation (e.g., method of genome extraction or library preparation), NGS platform, and other analysis tools. Nevertheless, the approach used for estimating the LOD in the present study is applicable not only to WES-AF but also to AFs measured by other NGS-based methods such as WGS [24]. Researchers must identify the LOD of the analytical method employed to ensure that it is suited to their research goals. The in-house LOD estimated by the approach as in this study would be useful for setting the cutoff threshold of AFs obtained by NGS.

## Conclusion

Second-generation sequencing, such as that used by the HiSeq 2500 sequencing system, and which was employed in this study, is a method that labels PCR-amplified DNA with a fluorescent molecule and detects the light emitted when the DNA fragment is extended [2]. This NGS device operates as a detector that reads fluorescent signals labeled at each base, and can therefore be considered an analytical device similar to many other spectroscopic instruments (e.g., fluorescence spectrophotometer and laser scanning microscope). Generally, it is important to evaluate the LOD during microanalysis in analytical chemistry. Particularly, in NGS-based analyses of genetic mutations, precise and accurate results must be provided by combining the wet analysis process (NGS-based analysis itself) and the dry bioinformatics process (e.g., type and purpose of gene mutation and results of AF). However, analysis processes differ depending on the purpose of analyzing genetic mutations, and it is therefore not easy to ensure analytical validity. Thus, as the LOD differs depending on the analysis system or platform used, it is extremely important to establish a system for detecting reliable signals for mutations with low AFs with high reproducibility and accuracy for each research purpose. In this study, we conducted a general performance test using a reference material with pre-validated specific mutations and confirmed the analytical validity for the LOD of the mutations. This method was used to estimate the LOD by repeatedly measuring the same genomic DNA sample under the same conditions and by examining variations in the error of the resultant AFs. For the various mutation analysis tools that have been previously developed, the respective algorithms used to determine LOD may not necessarily be disclosed. Therefore, it is useful and even necessary to determine or estimate the in-house LOD of AFs in NGS analysis based on actual measurements of reference material or biological material, as demonstrated in the present study.

## Methods

### Library preparation for whole-exome sequencing

WES was performed using a DNA sample (Quantitative Multiplex Reference Standard gDNA; Horizon Discovery, Cambridge, UK) with 20 mutations at 1.0–33.5% AF pre-validated by ddPCR. The concentration and total amount of nucleic acids required for WES analysis were determined by absorption spectrometry using a NanoDrop (Thermo Fisher Scientific, Waltham, MA, USA) and fluorescence analysis using the Quant-iT dsDNA BR Assay Kit (Thermo Fisher Scientific). The measured values obtained by these two analytical methods showed no deviation. The NGS workflow is summarized in Fig. 1. A total of 3 μg of DNA sample (reference material) was physically fragmented to several hundreds of base pairs (average of 250 bp) using Covaris Acoustic Solubilizer (Covaris, Inc., Woburn, MA, USA) according to the manufacturer’s protocol.

After fragmentation, the sheared DNA fragments were used to construct a library using SureSelect XT Human All Exon V5 and SureSelect XT Reagent Kit (Agilent Technologies, Santa Clara, CA, USA) according to the manufacturer’s protocol. Next, target exons were enriched using SureSelectXT Automated Target Enrichment for Illumina Paired-End Multiplexed Sequencing (Agilent Technologies). After enriched exome libraries were multiplexed, the libraries were sequenced using a HiSeq 2500 sequencing platform (Illumina, San Diego, CA, USA) as paired-end 100 base reads, according to the manufacturer’s protocol. Briefly, a paired-end DNA sequencing library was prepared by gDNA shearing, end-repair, A-tailing, paired-end adaptor ligation, and amplification. After hybridization of the library with bait sequences for 24 h, the captured library was purified and amplified with an index barcode tag. The size distribution and concentration of the library was determined with an Agilent Technologies 2100 Bioanalyzer.

Sequencing of the exome library was performed using the 100-bp paired-end mode of the TruSeq PE Cluster Kit v4 and TruSeq SBS Kit v4 (Illumina). The sequencing step described above was repeated four times per sample. The sequencing data and results are summarized in Table S2.

### Quality validation of sequence reads

We removed the 3′-end adaptor sequences with Cutadapt v1.2.1 (https://cutadapt.readthedocs.io/en/stable/) [25] with the set parameter -O 9 -m 32, and then removed the 3′-end of reads shorter than 32 bp as well as low-quality reads with fastq_quality trimmer (parameter: -l 32) and fastq_quality_filter (parameters: -q 10 -p 99) in FASTX-Toolkit v0.0.13 (http://hannonlab.cshl.edu/fastx_toolkit/). The cleaned paired reads were extracted using cmpfastq (http://compbio.brc.iop.kcl.ac.uk/software/cmpfastq_pe.php) in Cutadapt v1.2.1.

### Calculation of AF

Clean reads were mapped to the reference sequence, and the results of mapping were adjusted to conditions appropriate for mutation detection. First, clean reads were mapped to the UCSC hg19 reference genome (downloaded from http://genome.ucsc.edu) using bwa aln/sampe command with the default setting in BWA v0.5.9 (https://sourceforge.net/projects/bio-bwa/files/) [26]. Next, to generate sequencing data of varying sizes, downsampling of each mapping result was performed in Picard v1.93 (https://sourceforge.net/projects/picard/). Using the downsample command (java -jar DownsampleSam.jar I = inbam O = outbam P = [downsample ratio]) of Picard, the BAM file for each sample was downsampled to datasets of 5, 15, 30 and 40 Gbp based on the value of the downsample parameter. To correct suspected regions in the downsampled mapping data results, pre-calling was performed with GATK UnifiedGenotyper using default settings, followed by local realignment around indels by GATK RealignerTargetCreator/IndelRealigner in the Genome Analysis Toolkit (GATK) v1.6–9 (https://gatk.broadinstitute.org/hc/en-us) [27, 28]. Based on the realignment results, the sequences of reads aligned at each position were compared to the reference sequence, and the base quality was readjusted to a more accurate value by using GATK CountCovariates/IndelRealigner with the default setting. This step was performed based on the consistency between the reference and read sequences. The mutation base list (pre-calling) detected in the realigned results and known mutation data [e.g., The Single Nucleotide Polymorphism Database (dbSNP), if any] were used to identify regions where the reference and read sequences matched. From these mapping results, base types sequenced for 20 mutations were counted to calculate the AF using a custom script powered by Samtools v1.0 [29].

### Data analysis

Based on the analytical evidence, the LOD is characterized with 30%RSD [20–23]. To estimate the LOD of WES analysis, the %RSD was obtained for the AFs from four independent WES experiments. Next, the relationship between the mean AFs (mean of WES-AFs) obtained from quadruplicate technical library replicates and %RSD values of the mutations was graphed. However, the change in %RSD for the mean WES-AFs was not sufficient for capturing the overall trend; therefore, a moving average was used to smooth the data. Finally, LOD_30%RSD_ of WES analysis was estimated by reading the AF value of %RSD = 30% from the moving average curve of the %RSD value versus the mean value of WES-AFs.

## Supplementary Information

**Additional file 1: Figure S1.** Limit of detection (LOD) of allele frequency in whole-exome sequencing (WES) analysis. Three or five point-moving average curve plots of percentage relative standard deviation (%RSD) against the mean vales of WES-allele frequencies (AFs) were used. The %RSD values were plotted against the mean WES-AFs. The consistent trend in %RSD vs. mean WES-AFs is represented by the 3 or 5 point-moving average curve on the graph. The derivation of LOD_30%RSD_ is illustrated by dotted lines (see arrow). All analyses were performed using the following sequencing data sizes: 5 (A), 15 (B), 30 (C), and 40 (D) Gbp. **Figure S2.** AF LOD in WES analysis using the Illumina TruSeq Exome Enrichment Kit for library preparation. The Illumina TruSeq Exome Enrichment Kit was used to capture the exome region, and downstream analysis was performed using a workflow designed by Illumina, Inc. The %RSD values of AFs calculated from quadruplicate technical replicates were plotted against the mean values of AFs (mean WES-AFs) obtained from quadruplicate technical replicates. The consistent trend in %RSD vs. mean WES-AFs is represented by the moving average curve on the graph. The derivation of LOD_30%RSD_ is illustrated by dotted lines (see arrow). All analyses were performed using the following WES data sizes: 15 (A), 30 (B), and 40 (C) Gbp. (Note: the 5 Gbp WES data size was excluded from this analysis because the on-target rates at some low AF positions were very low.) (D) Line graph showing the trend in correlation between LOD and sequencing data size (from A–C). When WES was performed using a sequencing data size > 15 Gbp, the LOD was relatively constant and in the range of 5–10%. **Table S1.** Summary of sequencing quality (sequencing data size: approximately 5 – 40 Gbp). **Table S2.** Sequencing results. **Supplementary Methods.** Exome sequencing via the Illumina exome capture platform. Exome enrichment was independently performed with quadruplicate technical replicates using the TruSeq Exome Enrichment Kit (Illumina). After enriched exome libraries were multiplexed, the libraries were sequenced using a NextSeq 500 sequencing platform (Illumina) according to manufacturer’s instructions. We used the FASTQ Toolkit App in BaseSpace™ Sequence Hub designed by Illumina, Inc. to filter the data for quality and read length. Alignment to reference sequences and variant identification were performed with the Enrichment App (Illumina). The sequence data from this experiment has been deposited on the NCBI (BioProject accession number PRJNA670243).

## Data Availability

The raw sequence data for this study were submitted to NCBI (http://www.ncbi.nlm.nih.gov/) under the BioProject accession numbers PRJNA635795 and PRJNA670243.

## Abbreviations

AF: Allele frequency
Gbp: Giga base pairs
ddPCR: Digital droplet PCR
SNV: Single-nucleotide variants
WES: Whole-exome sequencing
WGS: Whole-genome sequencing
TS: Targeted sequencing
NGS: Next-generation sequencing
LOD: Limit of detection
%RSD: % relative standard deviation
dbSNP: The Single Nucleotide Polymorphism Database
