# Supplementary material for: A simple method to estimate the in-house limit of detection for genetic mutations with low allele frequencies in whole-exome sequencing analysis by next-generation sequencing
Source: BMC Genom Data. 2021 Feb 18;22:8. doi: 10.1186/s12863-020-00956-x (PMC7893872; doi:10.1186/s12863-020-00956-x)
Supplement: Supplementary file 1 — Additional file 1: Figure S1. Limit of detection (LOD) of allele frequency in whole-exome sequencing (WES) analysis. Three or five point-moving average curve plots of percentage relative standard deviation (%RSD) against the mean vales of WES-allele frequencies (AFs) were used. The %RSD values were plotted against the mean WES-AFs. The consistent trend in %RSD vs. mean WES-AFs is represented by the 3 or 5 point-moving average curve on the graph. The derivation of LOD30%RSD is illustrated by dotted lines (see arrow). All analyses were performed using the following sequencing data sizes: 5 (A), 15 (B), 30 (C), and 40 (D) Gbp. Figure S2. AF LOD in WES analysis using the Illumina TruSeq Exome Enrichment Kit for library preparation. The Illumina TruSeq Exome Enrichment Kit was used to capture the exome region, and downstream analysis was performed using a workflow designed by Illumina, Inc. The %RSD values of AFs calculated from quadruplicate technical replicates were plotted against the mean values of AFs (mean WES-AFs) obtained from quadruplicate technical replicates. The consistent trend in %RSD vs. mean WES-AFs is represented by the moving average curve on the graph. The derivation of LOD30%RSD is illustrated by dotted lines (see arrow). All analyses were performed using the following WES data sizes: 15 (A), 30 (B), and 40 (C) Gbp. (Note: the 5 Gbp WES data size was excluded from this analysis because the on-target rates at some low AF positions were very low.) (D) Line graph showing the trend in correlation between LOD and sequencing data size (from A–C). When WES was performed using a sequencing data size > 15 Gbp, the LOD was relatively constant and in the range of 5–10%. Table S1. Summary of sequencing quality (sequencing data size: approximately 5 – 40 Gbp). Table S2. Sequencing results. Supplementary Methods. Exome sequencing via the Illumina exome capture platform. Exome enrichment was independently performed with quadruplicate technical replicates using the T [file 12863_2020_956_MOESM1_ESM.zip › Supplementary Methods.docx]

**Supplementary Methods**

**Exome sequencing via the Illumina exome capture platform**

Exome enrichment was independently performed with quadruplicate technical replicates using the TruSeq Exome Enrichment Kit (Illumina). After enriched exome libraries were multiplexed, the libraries were sequenced using a NextSeq 500 sequencing platform (Illumina) according to manufacturer’s instructions. We used the FASTQ Toolkit App in BaseSpace™ Sequence Hub designed by Illumina, Inc. to filter the data for quality and read length. Alignment to reference sequences and variant identification were performed with the Enrichment App (Illumina). The sequence data from this experiment has been deposited on the NCBI (BioProject accession number PRJNA670243).
