# Supplementary material for: A simple method to estimate the in-house limit of detection for genetic mutations with low allele frequencies in whole-exome sequencing analysis by next-generation sequencing
Source: BMC Genom Data. 2021 Feb 18;22:8. doi: 10.1186/s12863-020-00956-x (PMC7893872; doi:10.1186/s12863-020-00956-x)
Supplement: Supplementary file 1 — Additional file 1: Figure S1. Limit of detection (LOD) of allele frequency in whole-exome sequencing (WES) analysis. Three or five point-moving average curve plots of percentage relative standard deviation (%RSD) against the mean vales of WES-allele frequencies (AFs) were used. The %RSD values were plotted against the mean WES-AFs. The consistent trend in %RSD vs. mean WES-AFs is represented by the 3 or 5 point-moving average curve on the graph. The derivation of LOD30%RSD is illustrated by dotted lines (see arrow). All analyses were performed using the following sequencing data sizes: 5 (A), 15 (B), 30 (C), and 40 (D) Gbp. Figure S2. AF LOD in WES analysis using the Illumina TruSeq Exome Enrichment Kit for library preparation. The Illumina TruSeq Exome Enrichment Kit was used to capture the exome region, and downstream analysis was performed using a workflow designed by Illumina, Inc. The %RSD values of AFs calculated from quadruplicate technical replicates were plotted against the mean values of AFs (mean WES-AFs) obtained from quadruplicate technical replicates. The consistent trend in %RSD vs. mean WES-AFs is represented by the moving average curve on the graph. The derivation of LOD30%RSD is illustrated by dotted lines (see arrow). All analyses were performed using the following WES data sizes: 15 (A), 30 (B), and 40 (C) Gbp. (Note: the 5 Gbp WES data size was excluded from this analysis because the on-target rates at some low AF positions were very low.) (D) Line graph showing the trend in correlation between LOD and sequencing data size (from A–C). When WES was performed using a sequencing data size > 15 Gbp, the LOD was relatively constant and in the range of 5–10%. Table S1. Summary of sequencing quality (sequencing data size: approximately 5 – 40 Gbp). Table S2. Sequencing results. Supplementary Methods. Exome sequencing via the Illumina exome capture platform. Exome enrichment was independently performed with quadruplicate technical replicates using the T [file 12863_2020_956_MOESM1_ESM.zip › Supplementary_data__revised_Miura_etal.pdf]

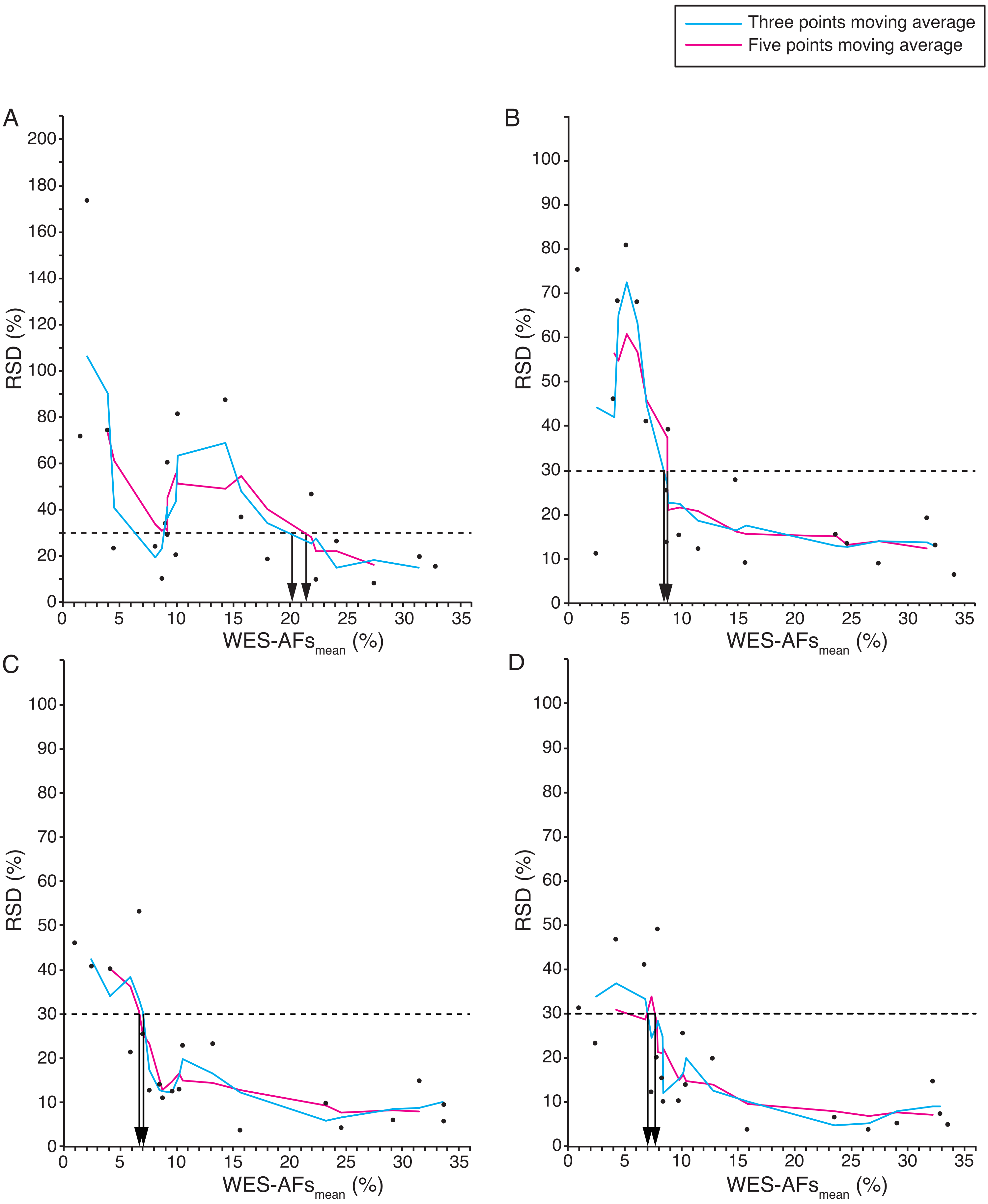
Three points moving average
Five points moving average
A
B
C
D
RSD (%)
WES-AFs$_{mean}$ (%)
0
5
10
15
20
25
30
35
20
40
60
80
100
120
140
160
180
200
30
50
70
90

Figure S1

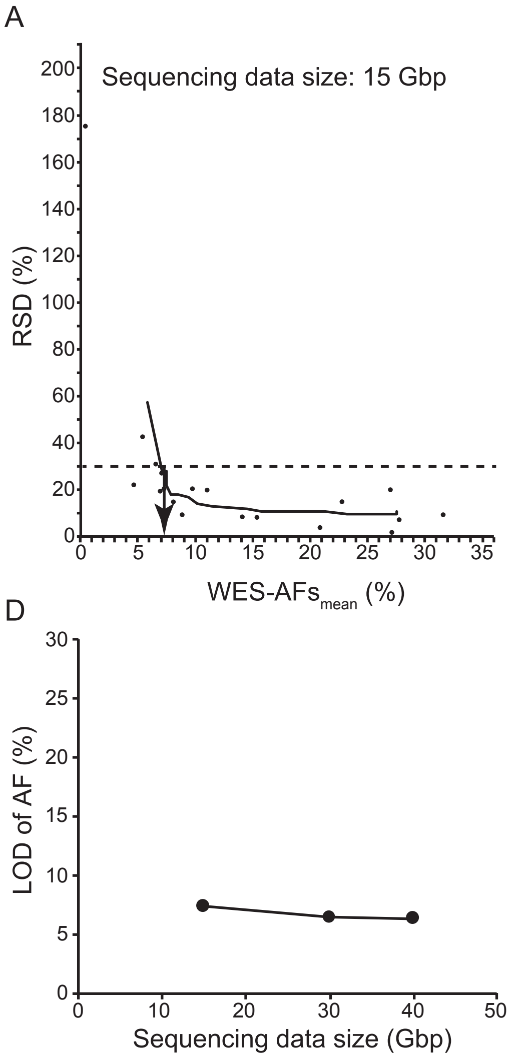

A
Sequencing data size: 15 Gbp
B
Sequencing data size: 30 Gbp
C
Sequencing data size: 40 Gbp
RSD (%)
WES-AFs$_{mean}$ (%)
D
LOD of AF (%)
Sequencing data size (Gbp)

Figure S2

Supplementary Table S1. Summary of sequencing quality (sequencing data size: approximately 5 – 40 Gbp)

Experimental run	Total amount of sequence data from mapped reads	Mean depth	Most frequent depth	Cover rate (%)				Total amount of sequence data from on-target reads	On-target rate of mapped reads (%)
				≥ 3×	≥ 10×	≥ 20×	≥ 40×		
Expt. 1 (5 Gbp)	50,129,796	62.21	39	99.59	97.80	91.16	67.94	37,940,862	75.83
Expt. 2 (5 Gbp)	50,152,376	63.60	37	99.62	97.85	91.47	68.76	38,573,426	77.16
Expt. 3 (5 Gbp)	50,028,568	63.87	37	99.56	97.70	91.26	68.59	38,774,039	77.73
Expt. 4 (5 Gbp)	50,022,468	62.16	37	99.59	97.73	91.05	67.84	37,892,712	75.97
Expt. 1 (15 Gbp)	149,601,442	186.02	108	99.83	99.59	99.01	96.47	113,436,127	75.83
Expt. 2 (15 Gbp)	149,438,384	190.12	107	99.83	99.61	99.03	96.56	115,311,369	77.16
Expt. 3 (15 Gbp)	149,635,407	191.58	115	99.82	99.56	98.91	96.39	116,312,306	77.73
Expt. 4 (15 Gbp)	149,633,891	186.47	108	99.83	99.59	98.97	96.37	113,660,296	75.96
Expt. 1 (30 Gbp)	299,168,460	371.97	218	99.87	99.78	99.60	99.03	226,829,923	75.82
Expt. 2 (30 Gbp)	298,865,617	380.24	224	99.87	99.79	99.62	99.05	230,620,899	77.17
Expt. 3 (30 Gbp)	299,272,629	383.16	226	99.86	99.76	99.56	98.94	232,622,051	77.73
Expt. 4 (30 Gbp)	299,308,203	372.98	222	99.87	99.78	99.60	99.00	227,347,314	75.96
Expt. 1 (40 Gbp)	398,707,137	495.73	291	99.88	99.82	99.70	99.37	302,300,883	75.82
Expt. 2 (40 Gbp)	398,814,205	507.42	308	99.87	99.82	99.71	99.38	307,757,783	77.17
Expt. 3 (40 Gbp)	399,028,211	510.86	300	99.87	99.80	99.67	99.29	310,151,890	77.73
Expt. 4 (40 Gbp)	398,617,453	496.71	287	99.87	99.81	99.70	99.34	302,770,752	75.96

On-target: calculation is conducted only if target regions exist. A read is assessed as an on-target read if at least one base overlaps a target region.

Supplementary Table S2. Sequencing results

Experimental run	Total reads	Total bases (bp)	Q30R1 (%)	Q30R2 (%)
Expt. 1	474,895,722	47,489,572,200	95.3	93.6
Expt. 2	529,964,716	52,996,471,600	94.8	92.0
Expt. 3	440,178,726	44,017,872,600	95.5	94.2
Expt. 4	469,051,582	46,905,158,200	95.0	93.5

Q30 : Phred quality score of 30
R1 : Forward read
R2 : Reverse read
